# Surface Properties of SnO_2_ Nanowires Deposited on Si Substrate Covered by Au Catalyst Studies by XPS, TDS and SEM

**DOI:** 10.3390/nano8090738

**Published:** 2018-09-18

**Authors:** Monika Kwoka, Barbara Lyson-Sypien, Anna Kulis, Dario Zappa, Elisabetta Comini

**Affiliations:** 1 Institute of Electronics, Silesian University of Technology, 44-100 Gliwice, Poland; Barbara.Lyson-Sypien@polsl.pl (B.L.-S.); Anna.Kulis@polsl.pl (A.K.); 2 SENSOR Lab, Department of Information Engineering (DII), Brescia University, 25123 Brescia, Italy; dario.zappa@unibs.it (D.Z.); elisabetta.comini@unibs.it (E.C.)

**Keywords:** tin dioxide SnO_2_ nanowires, vapor phase deposition, surface chemistry, surface morphology

## Abstract

The surface chemistry and the morphology of SnO_2_ nanowires of average length and diameter of several µm and around 100 nm, respectively, deposited by vapor phase deposition (VPD) method on Au-covered Si substrate, were studied before and after subsequent air exposure. For this purpose, surface-sensitive methods, including X-ray photoelectron spectroscopy (XPS), thermal desorption spectroscopy (TDS) and the scanning electron microscopy (SEM), were applied. The studies presented within this paper allowed to determine their surface non-stoichiometry combined with the presence of carbon contaminations, in a good correlation with their surface morphology. The relative concentrations of the main components [O]/[Sn]; [C]/[Sn]; [Au]/[Sn], together with the O–Sn; O–Si bonds, were analyzed. The results of TDS remained in a good agreement with the observations from XPS. Moreover, conclusions obtained for SnO_2_ nanowires deposited with the use of Au catalyst were compared to the previous obtained for Ag-assisted tin dioxide nanowires. The information obtained within these studies is of a great importance for the potential application of SnO_2_ nanowires in the field of novel chemical nanosensor devices, since the results can provide an interpretation of how aging effects influence gas sensor dynamic characteristics.

## 1. Introduction

Among transparent conductive oxide (TCO) semiconductors applied to the gas sensor devices [1], flat-panel displays [2,3] and solar cells [4], tin dioxide still remains one of the leading compounds under investigation (together with In_2_O_3_ and ZnO) due to its unique features, such as: high electrical conductivity (~10^2^ Ω^−1^·cm^−1^) [5], a wide band gap (3.6 eV) [6] and an average transparency in the visible and near infrared region at the level of 80%.

Recently, there has been a continuously increasing tendency to fabricate new forms of SnO_2_ starting from thick and thin films [7,8,9] to nano-scale objects such as: nanowires [10,11], nanofibers [12], nanopowders [13], and nanorods [14], with the aim of obtaining enhanced and tuned features. In the field of gas sensors, the improved sensitivity, selectivity, and thermal stability, as well as the speed of response and recovery are the most desired characteristics that can be achieved using nanowires [15,16]. This can be attributed to the high crystallinity, less agglomerated structures’ formation as well as the large surface-to-volume ratio. In such structures, there is a crucial role (domination) of the atoms localized just at the surface [17], where the sensor transduction mechanism takes place, and therefore leading to the enhancement of the chemical sensing performance (such as catalytic activity or surface adsorption) [18,19].

The development in the field of SnO_2_-based nanowires tends toward intentionally applying not only other metal oxide additives but also noble metal catalysts such as Pd [19,20,21], Pt [21,22], Ag [10,23], and Au [12,24] in order to both induce nucleation of the nanowires [10] and to achieve an improvement in the sensor signal, operating temperature, and stability to humidity [7]. Despite the great efforts undertaken worldwide to reach simultaneously all the features listed above, it appears that there is still much that can be done to optimize properties of SnO_2_ based nanowires, as they strongly depend on the deviation from stoichiometry, the amount of dopants/impurities, and the microstructure, being at the same time strongly influenced by the undesired surface contaminations.

Driven by this requirement, in our previous work [10], we have investigated the surface chemistry of SnO_2_ nanowires deposited on Ag-covered Si substrate by vapor phase deposition (VPD) method using X-ray photoelectron spectroscopy (XPS), in combination with thermal desorption spectroscopy (TDS), with the special emphasis on identifying and avoiding carbon contaminations at the surface that are directly related to the aging effects.

In this work we undertake the issue of avoiding/removing surface contamination in the case of SnO_2_ based nanowires grown on Au-assisted substrate, as it has been reported that gold can significantly improve the sensing properties of tin dioxide films due to the increased concentration of oxygen species [7]. We present the results of comparative studies of the surface chemistry and morphology of SnO_2_ nanowires prepared by VPD method on Au-covered Si substrate after air exposure. The VPD technique is chosen since it is considered as the most successful method for the deposition of SnO_2_ nanowires on the Si surface covered with an ultrathin layer of metallic catalyst in the form of metallic droplets (metallic seeds), which play a crucial role for the nucleation and growth of SnO_2_ nanowires from the gaseous reactants [25]. Moreover, it enables the control of SnO_2_ nanowires’ size and their density at the substrate [26].

Au-assisted SnO_2_ nanostructures exhibit different properties when compared to Pd or Pt-SnO_2_ systems (Fermi-level control behavior). As there are reports that neither surface nor bulk interactions take place between Au and SnO_2_ [7], the surface chemistry of these nanostructures still needs further investigation and analysis. In order to design a novel gas sensor based on SnO_2_ nanowires deposited on Au-coated substrate, which can be treated as the long-term perspective for the results of these studies, the knowledge concerning the surface chemistry of this system is crucial.

## 2. Experimental

### 2.1. Deposition of SnO_2_ Nanowires

SnO_2_ nanowires were synthesized at SENSOR Lab, Department of Information Engineering (DII), University of Brescia, Italy. The process started with the cleaning of a Si (100) wafer in acetone for 15 min. After that procedure, the substrate was covered with an ultrathin Au layer (5 nm) deposited by magnetron sputtering (MS) with a radio frequency (RF) electrical power of 50 W and 7 sccm Ar flow (Kenotec, Italy). The methodology described above was applied to improve the SnO_2_ nanowires nucleation process, since Au metallic seeds act as nucleation sites that promote the growth of SnO_2_ nanowires in the VPD [26].

The deposition of SnO_2_ nanowires was performed in a tubular alumina furnace (custom design, based on a Lenton furnace), using SnO_2_ powder (99.9% purity, Sigma-Aldrich, St. Louis, MO, USA) as a source material and heated up to 1370 °C in order to start the evaporation process. The mass transport was achieved by introducing an argon flow acting as gas carrier at a pressure of 100 mbar. After 15 min, a dense and homogeneous mat of SnO_2_ nanowires was obtained on the Si substrate covered with Au nanolayers held at a temperature of 900 °C.

### 2.2. Characterization of SnO_2_ Nanowires

The surface morphology of SnO_2_ nanowires (NW) was controlled using a field emission scanning electron microscope (FE-SEM, Gemini, Leo 1525, One Zeiss Drive, Thornwood, NY, USA). The measurements were carried out at SENSOR Lab at the University of Brescia in Italy. These observations confirmed the nanostructures’ size and morphology.

In turn, the surface chemistry, including contaminations, of the above mentioned SnO_2_ NW was controlled sequentially using XPS in combination with the TDS method. The XPS technique enabled the determination of both the surface stoichiometry and the contaminations before and after the TDS experiments, which allowed us to detect the active gases adsorbed at the surface of SnO_2_ NW after air exposure. The TDS procedure consisted of measuring gas desorption during the programmable linear growth of the sample temperature (TPD) detected in line by mass spectrometry (MS, model Stanford RGA200, Sunnyvale, CA, USA). These experiments were done at the CESIS Centre, Institute of Electronics, Silesian University of Technology, Gliwice, Poland.

In X-ray photoelectron spectroscopy experiments, the XPS spectrometer (SPECS, Berlin, Germany) was equipped with a X-ray lamp (AlK_α_ 1486.6 eV; XR-50 model) and a concentric hemispherical analyzer (PHOIBOS-100 Model). All the reported binding energy (BE) data was calibrated using the XPS C1s peak (285.0 eV) of residual C contamination at the surface of SnO_2_ nanowires. The XPS experiments were performed at the base background working pressure ~10^−7^ Pa. More details concerning the experimental setup can be found in [10,27].

TDS measurements were performed also at the base background working pressure of ~10^−7^ Pa in the sample preparation chamber equipped, among others, with a mass spectrometer-residual gas analyzer RGA100 Model (Stanford Research System, Sunnyvale, CA, USA) and a temperature programmable control unit of OmniVac-Dual Regulated Power Supply PSReg120 model. During each TPD cycle (the temperature increased by 6 °C per minute in the range of 50–350 °C) the TDS spectra of selected gases like H_2_, H_2_O, O_2_, and CO_2_ have been registered.

## 3. Results and Discussion

Crystalline properties of SnO_2_ nanowires deposited on various substrates investigated by XRD were described in a set of our recent papers [26,28,29]. Within these studies, only a pure cassiterite crystal structure was observed for all tin oxide nanowires under investigation.

**Figure 1:**
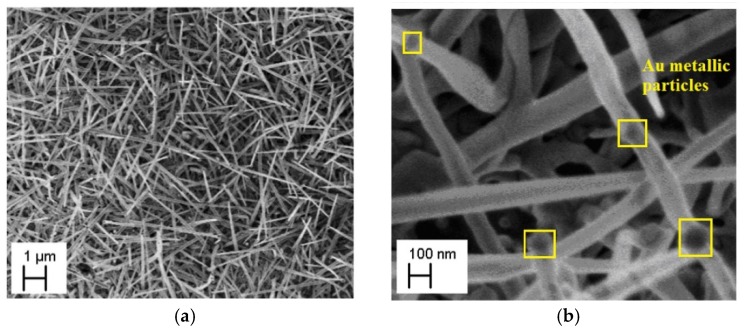
The scanning electron microscopy (SEM) images (in two scales (**a**) 1 µm and (**b**) 100 nm) of vapor phase deposition (VPD) SnO_2_ nanowires deposited on Au-covered Si (100) substrate.

Figure 1 presents SEM images of VPD SnO_2_ nanowires deposited on Au-covered Si (100) substrate. As it can be seen, the nanowires are isolated, and their lengths were equal to several µm. The diameter, instead, was around 100 nm. Au metallic nanoparticles that had migrated from the substrate and contributed to the SnO_2_ nanowire nucleation process, were easily identified on the surface of the nanostructures.

**Figure 2:**
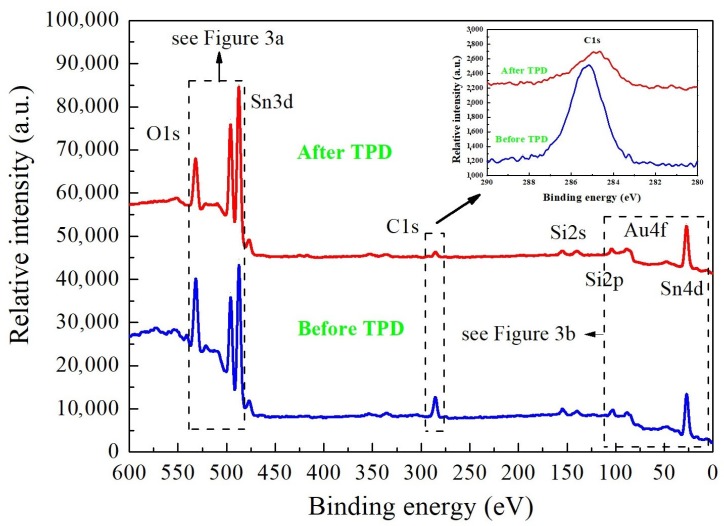
X-ray photoelectron spectroscopy (XPS) survey spectra with main core level lines of the VPD SnO_2_ nanowires deposited on Au-covered Si substrate before and after the programmable linear growth of the sample temperature (TPD) process.

Figure 2 shows the XPS survey spectra of the VPD SnO_2_ nanowires deposited on Au-covered Si substrate after exposure to air, before and after the TPD process.

The spectra contain well-recognized main core XPS levels: O1s, double Sn3d, single C1s, and Sn4d peaks, correspondingly labelled. The evident contribution of the XPS C1s peak can be attributed to the carbon contamination adsorbed at the surface from the air atmosphere. Moreover, the Au4f peaks are present, proving that the metallic Au catalyst appeared at the surface of nanowires. This conclusion is opposite to our recent experience, as the presence of the Ag catalyst was not observed in VPD SnO_2_ nanowires deposited on Ag-covered Si (100) substrate [10].

The inset in Figure 2 shows in detail the binding energy range corresponding to the C1s peaks position. As it can be seen, the removal of carbon contaminations during the TDS procedure is not completely possible. Relying on the SEM (Figure 1) as well as XPS results before and after TDS procedure (Figure 2), it can be concluded that the gold particles were covered by the carbon, and the bonding between them impeded the removal of C contamination during the TDS process. This is a limitation that has not been observed in the case of the previously studied Ag-covered Si (100) substrate [10].

Quantitative XPS data analysis was performed to determine the relative concentration of the main components of the SnO_2_ nanowires under investigation within the escape depth, corresponding to the triple values of the inelastic mean free path of the photoelectrons.

**Figure 3:**
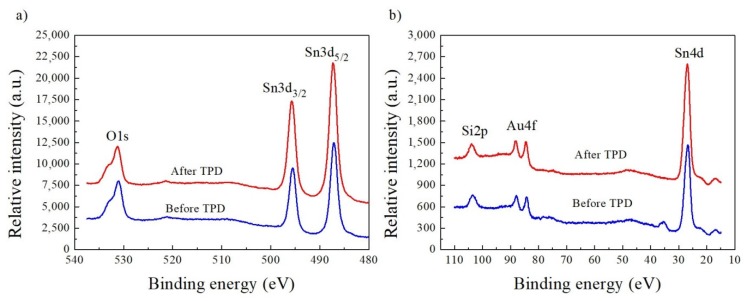
XPS spectral window of: (**a**) O1s and Sn3d, (**b**) Si2p, Au4f, and Sn4d of the VPD SnO_2_ nanowires deposited on Au-covered Si (100) substrate before and after the subsequent thermal desorption spectroscopy (TDS) experiment.

According to the generally accepted procedure, the relative concentration of the main components can be estimated on the base of the area (intensity) of the main XPS spectral lines within specific spectral windows, acquired with the highest signal-to-noise ratio (S/N) weighted by the corresponding atomic sensitivity factor (ASF) [30]. The details of this procedure were already described in reference [30]. Figure 3 demonstrates the XPS-specific spectral windows used for this procedure.

Taking into account the atomic sensitivity factors for respective elements (O1s-0.711 and Sn3d_5/2_-4.725) [27] the relative atomic concentrations [O]/[Sn] were calculated. After the air exposure, the relative [O]/[Sn] concentration at the surface of VPD SnO_2_ nanowires deposited at Au-covered Si (100) substrate reached a value of 2.26 ± 0.05. This means that the additional surface oxygen bonding appeared. In turn, after the TPD process, the relative [O]/[Sn] concentration decreased to 1.84 ± 0.05. This was evidently contrary to the results that were obtained for VPD SnO_2_ nanowires deposited on Ag-covered Si (100) substrate [10]. Perhaps, it corresponded to the deterioration of their stoichiometry, related to the desorption of weakly (physically) bounded oxygen from the surface of VPD SnO_2_ nanowires deposited on Au-covered Si (100) substrate.

Since evident XPS Au4f peaks were observed at the surface of VPD SnO_2_ nanowires, both before and after the TDS process (Figure 3b), the same procedure was applied to determine the [Au]/[Sn] ratio. The atomic sensitivity factor for Au4f equals to 6.250 [30]. The relative [Au]/[Sn] concentration before and after the TPD process appeared to be constant and equaled to 0.11.

Keeping in mind that C contamination at the surface of NWs is crucial, which was confirmed by C1s XPS peaks in the survey spectrum in Figure 2, the same procedure was used to determine [C]/[Sn] concentration. The atomic sensitivity factor for C1s is equal to 0.296 [30]. For VPD SnO_2_ nanowires deposited on Au-covered Si (100) substrate after air exposure, the relative [C]/[Sn] concentration was estimated as 2.66 ± 0.05, confirming that their surfaces are very strongly contaminated by C species. In turn, after the TDS process, the relative [C]/[Sn] concentration decreased more than five times, to the value of 0.54 ± 0.05.

The relative concentrations of [O]/[Sn], [C]/[Sn] and [Au]/[Sn] for VPD SnO_2_ nanowires deposited on Au-covered Si (100) substrate before and after TDS experiments are summarized in Table 1.

The variation of relative [O]/[Sn] concentration before and after TDS experiments, summarized in Table 1, is in good correlation with the variation of the contribution of the respective surface bonds of main elements, observed after the deconvolution of O1s and Sn3d_5/2_ XPS spectral lines (peaks).

**Figure 4:**
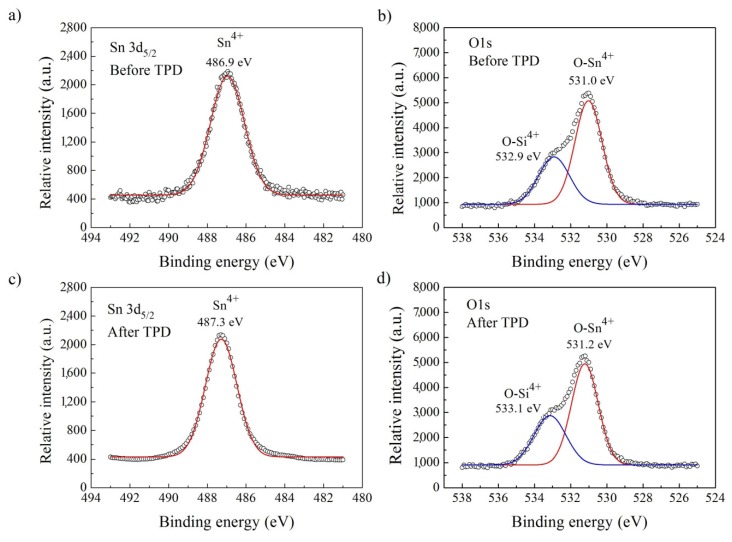
Registered XPS Sn3d_5/2_ and O1s peaks, and deconvoluted components of the air-exposed VPD SnO_2_ nanowires: (**a**,**b**) before TDS; (**c**,**d**) after the TDS process.

In Figure 4a,c, the XPS Sn3d_5/2_ lines of VPD SnO_2_ nanowires before and after TDS are presented. A simple visual shape analysis indicates that they are almost symmetrical. Thus, it is evident that they should contain only the component corresponding to one form of the Sn ions.

The decomposition of the Sn3d_5/2_ peaks allows us to conclude that there is only one contribution observed for both Sn3d_5/2_ lines (before and after TDS), corresponding to the Sn^4+^ ions, in good agreement with the National Institute of Standards and Technology (NIST) database [31]. Therefore, this confirms that our VPD SnO_2_ nanowires consisted mainly of tin dioxide SnO_2_. This was expected as they have been deposited directly starting from pure SnO_2_ powder.

In Figure 4b,d, XPS O1s lines of air-exposed VPD SnO_2_ nanowires are presented. Visual shape analysis indicates that they are evidently asymmetrical; thus, they should contain a minimum of two components corresponding to the various forms of oxygen ions. The decomposition of XPS O1s peaks shows that they are mainly built up of two contributions observed at the binding energies of: 531.0 eV and 531.2 eV, as well as 532.9 eV and 533.1 eV for the samples, before and after the TDS procedure, respectively.

The first binding energy can be ascribed to the oxygen bonds with the Sn^4+^ ions, whereas the second one to the oxygen bonds with the Si^4+^ ions of the substrate, in good agreement with the NIST database [31]. Therefore, in XPS experiments, we observed a contribution from the Si substrate, meaning that the whole Si substrate surface was not fully covered with VPD SnO_2_ nanowires.

The results of the decomposition procedure of XPS Sn3d_5/2_ and O1s peaks obtained for VPD SnO_2_ nanowires deposited on Au-covered Si substrate before and after TDS are summarized in Table 2.

Based on the information in Table 2, only a fraction of measured oxygen was effectively bonded with Sn^4+^ tin. Taking into account the contribution from these O–Sn^4+^ bonds, the real [O]/[Sn] concentration for VPD SnO_2_ nanowires before TDS process is smaller than the one obtained from the analysis based on the XPS O1s and Sn3d_5/2_ spectral windows (presented in Table 1). It means that these VPD SnO_2_ nanowires deposited on Au-covered Si substrate were not fully stoichiometric both before and after TDS.

The fact that the deconvolution procedure leads to the same conclusions for SnO_2_ nanowires before and after TDS, means that the surface chemistry of these nanostructures is practically stable. However, the exact value of the relative [O]/[Sn] concentration before and after the TDS process is not clear because of the contribution of the surface contaminations.

**Figure 5:**
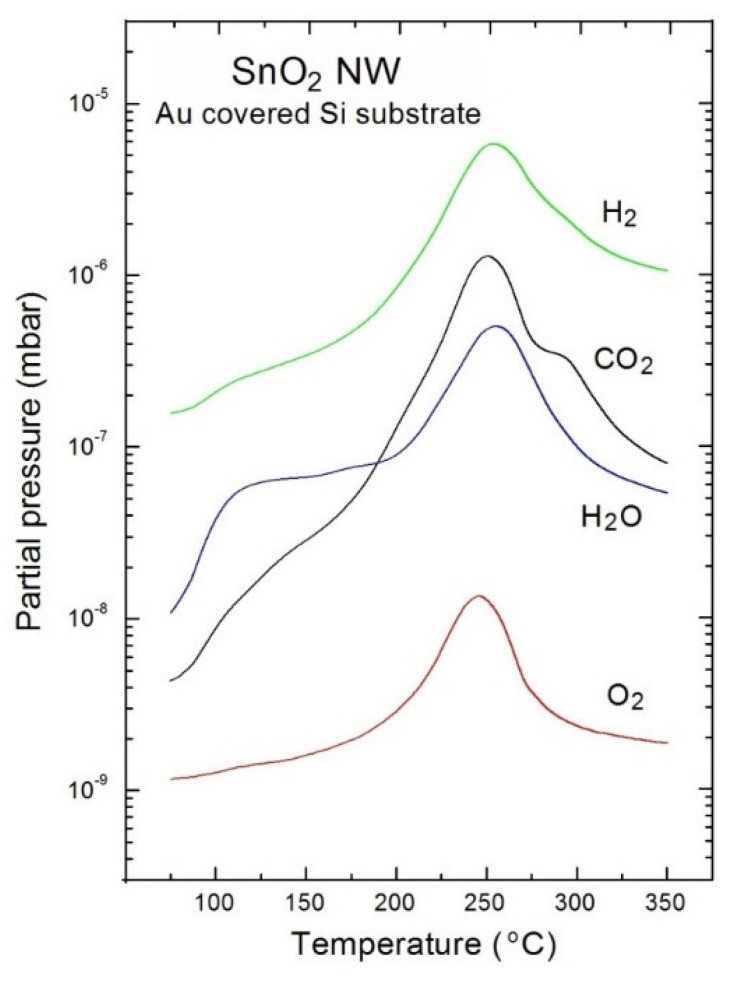
TDS spectra of the main residual gases desorbed from the VPD SnO_2_ nanowires deposited on an Au-covered Si substrate.

All obtained information on the evolution of surface chemistry of VPD SnO_2_ nanowires, before and after the TPD process, remains in a good correlation with the respective TDS spectra after smoothing (according to the typical procedure commonly used in the literature), as shown in Figure 5.

One can easily note that the molecular oxygen (O_2_) desorbs from VPD SnO_2_ nanowires at the partial pressure of about 10^−8^ mbar at 240 °C (maximum of the mass peak at this temperature). It means that the oxygen is probably only partially bounded to Sn, creating the SnO_2_ forms, and that there is a small amount of residual oxygen, which is physically bounded to the surface of the VPD SnO_2_ nanowires, that can easily desorb during the TDS experiment. This conclusion from the TDS spectra corresponds to a decrease of [O]/[Sn] concentration after thermal desorption, as was shown in XPS measurements.

In turn, molecular hydrogen desorbs during the TPD process at the highest partial pressure of 10^−5^ mbar, with a maximum at 240 °C. This last observation is also probably related to the single crystalline form of SnO_2_ nanowires [28], as in previously analysed VPD SnO_2_ nanowires. This is because molecular hydrogen exhibits a tendency to deeply penetrate the subsurface crystalline region [29], and in this case, also inside Au clusters, combined with fast and strong desorption during TDS experiments.

For the case of water vapor (H_2_O), the maximum partial pressure was observed at 10^−6^ mbar, at about 260 °C. What is even more important, the fastest desorption of H_2_O was seen at a temperature that was close to 100 °C.

The most important TPD effect was observed for carbon dioxide (CO_2_). The respective TDS spectrum exhibited a more complicated asymmetric and irregular shape over a temperature range of 250–320 °C. This probably means that the C-containing surface contaminations were bound in two different forms, and with different bonding energies at the external surface of the crystalline VPD SnO_2_ nanowires.

These last observations related to the desorption behavior of O_2_ and H_2_O were in a good correlation with the decrease of the relative [O]/[Sn] concentration observed by XPS (see Figure 3). In turn, the desorption behavior of CO_2_ explains the removal of C contaminations from these VPD SnO_2_ nanowires (see XPS C1s spectra in Figure 2).

The information presented above on the surface non-stoichiometry and surface C contaminations of VPD SnO_2_ nanowires is extremely important because of two reasons:

(1) The evident surface non-stoichiometry observed for VPD SnO_2_ nanowires causes their improved gas sensitivity, especially for oxidizing gases, with respect to the commonly used two-dimensional (2D) nanostructures [16].

(2) The existence of surface C contaminations at the surface, observed even after the TDS process, can play an undesired role acting as a specific barrier for the gas adsorption during the gas sensing process, and causing, among others, longer response and recovery times. This may be an evident limitation of their potential application for gas sensors systems.

## 4. Conclusions

The comparative investigation of VPD SnO_2_ nanowires deposited on Au-covered Si substrate using XPS, TDS, and SEM methods showed that:

(1) SnO_2_ nanowires exhibit a slight deviation from stoichiometry both before and after TDS. However, the presence of oxygen vacancy defects can lead to improved gas sensitivity, especially for oxidizing gases.

(2) Carbon contamination appears on the surface of SnO_2_ nanostructures. Its contribution decreases after the TDS procedure; however, it cannot be completely removed, unlike in the corresponding experiments performed on Ag-assisted SnO_2_ nanowires.

(3) Au metallic particles present on the surface of nanowires impede carbon desorption during TDS.

(4) All other active gases, physically adsorbed at the surface of VPD SnO_2_ nanowires after atmosphere exposure and creating surface contaminations, were almost fully desorbed during TDS.

(5) Based on the results of the deconvolution procedure, it can be concluded that the surface chemistry of the VPD SnO_2_ nanowires was stable.

## Figures and Tables

**Table 1:** The relative atomic ratios of basic elements for VPD SnO_2_ nanowires deposited on Au-covered Si (100) substrate.

Sample Status	Relative Concentration		
	[O]/[Sn]	[C]/[Sn]	[Au]/[Sn]
**Before TPD**	2.26 ± 0.05	2.66 ± 0.05	0.11 ± 0.05
**After TPD**	1.84 ± 0.05	0.54 ± 0.05	0.11 ± 0.05

**Table 2:** The basic parameters of XPS Sn3d_5/2_ and O1s peaks used in the deconvolution procedure and the obtained contribution of the main components for the samples both before as well as after TDS.

	Before TDS			After TDS		
**XPS Peak Parameters**	Sn3d_5/2_	O1s		Sn3d_5/2_	O1s	
**Components**	Sn^4+^	O–Sn^4+^	O–Si^4+^	Sn^4^	O–Sn^4+^	O–Si^4+^
**Binding Energy (eV)**	486.9	531.0	532.9	487.3	531.2	533.1
**Relative Peak Area**	1.0	0.66	0.34	1.0	0.63	0.37

## References

[1] Göpel W., Schierbaum K.D. (1995). SnO_2_ sensors: current status and future prospects. Sens. Actuators B Chem..

[2] Lewis B.G., Paine D.C. (2000). Applications and processing of transparent conducting oxides. MRS Bull..

[3] Goncalves G., Grasso V., Barquinha P., Pereira L., Elamurugu E., Brignone M., Martins R., Lambertini V., Fortunato E. (2011). Role of room temperature sputtered high conductive and high transparent indium zinc oxide film contacts on the performance of orange, green, and blue organic light emitting diodes. Plasma Process. Polym..

[4] Dewar A.L., Jain A.K., Jagadish C., Hartnagel H. (1995). Semiconducting Transparent Thin Films.

[5] Park S., Zheng H., Mackenzie J.D. (1995). Sol-gel derived antimony-doped tin oxide coatings on ceramic cloths. Mater. Lett..

[6] Khan A., Mehmood M., Aslam M., Ashraf M. (2010). Characteristics of electron beam evaporated nanocrystalline SnO_2_ thin films annealed in air. Appl. Surf. Sci..

[7] Hübner M., Koziej D., Grunwaldt J.-D., Weimar U., Barsan N. (2012). An Au clusters related spill-over sensitization mechanism in SnO_2_-based gas sensors identified by operando HERFD-XAS, work function changes, DC resistance and catalytic conversion studies. Phys. Chem. Chem. Phys..

[8] Barsan N., Hübner M., Weimar U. (2011). Conduction mechanism in SnO_2_ based polycrystalline thick film gas sensors exposed to CO and H_2_ in different oxygen background. Sens. Actuators B Chem..

[9] Yamazoe N., Shimanoe K. (2003). Oxide semiconductor gas sensors. Catal. Surv. Asia.

[10] Sitarz M., Kwoka M., Zappa D., Comini E., Szuber J. (2014). Surface chemistry of SnO_2_ nanowires on Ag-catalyst-covered Si substrate studied using XPS and TDS methods. Nanoscale Res. Lett..

[11] Katoch A., Sun G.-J., Choi S.-W., Hishita S., Kulish V.V., Wu P., Kim S. (2014). Acceptor-compensated charge transport and surface chemical reactions in Au-implanted SnO_2_ nanowires. Sci. Rep..

[12] Park J.Y., Asokan K., Choi S.-W., Kim S.S. (2011). Growth kinetics of nanograins in SnO_2_ fibers and size dependent sensing properties. Sens. Actuators B Chem..

[13] Lyson-Sypien B., Czapla A., Lubecka M., Kusior E., Zakrzewska K., Radecka M., Kusior A., Balogh A.G., Lauterbach S., Kleebe H.-J. (2013). Gas sensing properties of TiO_2_-SnO_2_ nanomaterials. Sens. Actuators B Chem..

[14] Choi S.-W., Katoch A., Sun G.-J., Wu P., Kim S.S. (2013). NO_2_-sensing performance of SnO_2_ microrods by functionalization of Ag nanoparticles. J. Mater. Chem. C.

[15] Comini E. (2006). Metal oxide nano-crystals for gas sensing. Anal. Chim. Acta.

[16] Kolmakov A., Moskovits M. (2004). Chemical sensing and catalysis by one-dimensional metal oxide nanostructures. Annu. Rev. Mater. Res..

[17] Wang B., Zhu L.F., Yang Y.H., Xu N.S., Yang G.W. (2008). Fabrication of a SnO_2_ Nanowire Gas Sensor and Sensor Performance for Hydrogen. J. Phys. Chem. C.

[18] Yamazoe N., Shimanoe K. (2011). Basic approach to the transducer function of oxide semiconductor gas sensors. Sens. Actuators B Chem..

[19] Kolmakov A., Klenov D.O., Lilach Y., Stemmer S., Moskovits M. (2005). Enhanced gas sensing by individual SnO_2_ nanowires and nanobelts functionalized with Pd catalyst particles. Nano Lett..

[20] Shen Y., Yamazaki T., Liu Z.F., Meng D., Kikuta T., Nakatani N., Saito M., Mori M. (2009). Microstructure and H_2_ gas sensing properties of undoped and Pd-doped SnO_2_ nanowires. Sens. Actuators B Chem..

[21] Choi S.-W., Katoch A., Sun G.-J., Kim S.S. (2013). Bimetallic Pd/Pt nanoparticle-functionalized SnO_2_ nanowires for fast response and recovery to NO_2_. Sens. Actuators B Chem..

[22] Shafiei M., Kalantar-Zadeh K., Wlodarski W., Comini E., Ferroni M., Sberveglieri G., Kaciulis S., Pandolfi L. (2008). Hydrogen gas sensing performance of Pt/SnO_2_ nanowires/SiC MOS devices. Int. J. Smart Sensing Intell. Syst..

[23] Hwang I.-S., Choi J.-K., Woo H.-S., Kim S.-J., Jung S.-Y., Seong T.-Y., Kim I.-D., Lee J.-H. (2011). Facile control of C_2_H_5_OH sensing characteristics by decorating discrete Ag nanoclusters on SnO_2_ nonowire networks. ACS Appl. Mater. Interfaces.

[24] Yin W., Wei B., Hu C. (2009). In situ growth of SnO_2_ nanowires on the surface of Au-coated Sn grains using water-assisted chemical vapor deposition. Chem. Phys. Lett..

[25] Xia Y., Yang P., Sun Y., Wu Y., Mayers B., Gates B., Yin Y., Kim F., Yan H. (2003). One-dimensional nanostructures: synthesis, characterization and applications. Adv. Mater..

[26] Sberveglieri G., Baratto C., Comini E., Faglia G., Ferroni M., Pardo M., Ponzoni A., Vomiero A. (2009). Semiconducting tin oxide nanowires and thin films for chemical warfare agents detection. Thin Solid Films.

[27] Kwoka M., Ottaviano L., Koscielniak P., Szuber J. (2014). XPS, TDS, and AFM studies of surface chemistry and morphology of Ag-covered L-CVD SnO_2_ nanolayers. Nanoscale Res. Lett..

[28] Comini E., Baratto C., Faglia G., Ferroni M., Vomiero A., Sberveglieri G. (2009). Quasi-one dimensional metal oxide semiconductors: Preparation, characterization and application as chemical sensors. Prog. Mater. Sci..

[29] Comini E., Faglia G., Ferroni M., Ponzoni A., Vomiero A., Sberveglieri G. (2009). Metal oxide nanowires: Preparation and application in gas sensing. J. Mol. Catal. A Chem..

[30] Wagner C.D., Riggs W.M., Davis L.E., Moulder J.F., Mnilenberger G.E. (1979). Handbook of Xray Photoelectron Spectroscopy.

[31] NIST X-ray Photoelectron Spectroscopy Database. http://srdata.nist.gov/xps/.

